# Future enhanced clinical role of pharmacists in Emergency Departments in England: multi-site observational evaluation

**DOI:** 10.1007/s11096-017-0497-4

**Published:** 2017-06-26

**Authors:** Elizabeth Hughes, David Terry, Chi Huynh, Konstantinos Petridis, Matthew Aiello, Louis Mazard, Hirminder Ubhi, Alex Terry, Keith Wilson, Anthony Sinclair

**Affiliations:** 1 Health Education England - St Chads Court, 213 Hagley Road, Edgbaston, West Midlands, Birmingham, B16 9RG UK; 2 Academic Practice Unit, Pharmacy Department, Birmingham Women’s and Children’s Hospital NHS Foundation Trust, Steelhouse Lane, Birmingham, B4 6NH UK; 3 Pharmacy Department, School of Life and Health Sciences, Aston University, Aston Triangle, Birmingham, B4 7ET UK; 4 Aston Business School, Aston University, Aston Triangle, Birmingham, B4 7ET UK; 5 Pharmacy Department, Birmingham Women’s and Children’s Hospital NHS Foundation Trust, Steelhouse Lane, Birmingham, B4 6NH UK

**Keywords:** Clinical pharmacy, Emergency Department, Pharmacist, Pharmacist training

## Abstract

*Background* There are concerns about maintaining appropriate clinical staffing levels in Emergency Departments. Pharmacists may be one possible solution. *Objective* To determine if Emergency Department attendees could be clinically managed by pharmacists with or without advanced clinical practice training. *Setting* Prospective 49 site cross-sectional observational study of patients attending Emergency Departments in England. *Method* Pharmacist data collectors identified patient attendance at their Emergency Department, recorded anonymized details of 400 cases and categorized each into one of four possible options: cases which could be managed by a community pharmacist; could be managed by a hospital pharmacist independent prescriber; could be managed by a hospital pharmacist independent prescriber with additional clinical training; or medical team only (unsuitable for pharmacists to manage). Impact indices sensitive to both workload and proportion of pharmacist manageable cases were calculated for each clinical group. *Main outcome measure* Proportion of cases which could be managed by a pharmacist. *Results* 18,613 cases were observed from 49 sites. 726 (3.9%) of cases were judged suitable for clinical management by community pharmacists, 719 (3.9%) by pharmacist prescribers, 5202 (27.9%) by pharmacist prescribers with further training, and 11,966 (64.3%) for medical team only. Impact Indices of the most frequent clinical groupings were general medicine (13.18) and orthopaedics (9.69). *Conclusion* The proportion of Emergency Department cases that could potentially be managed by a pharmacist was 36%. Greatest potential for pharmacist management was in general medicine and orthopaedics (usually minor trauma). Findings support the case for extending the clinical role of pharmacists.

## Impacts of practice

- Advanced trained clinical pharmacists may support the clinical workload of Emergency Departments.
- Up to 8% of patients attending Emergency Departments may be clinically managed by pharmacists with existing training, and a further 28% after additional clinical training.
- To ensure the highest impact on clinical workload advanced training of pharmacists should focus on general medicine and orthopaedics (usually minor trauma).

## Introduction

### Background

At present there are concerns about maintaining appropriate clinical staffing levels in Emergency Departments (ED) in England [1] and in other countries [2, 3]. One possible solution is the extension of clinical activity performed by non-medical staff—including pharmacists [4]. Subsidiary clinical management of ED attendees may support patient through-put, relieve pressure on medical staff and reduce costs.

Extending the pharmacist’s role in ED may also contribute to error minimization [3, 5]. Publications concerning pharmacists working within ED are usually focused on drug management, their role in ‘Rapid Response Teams’ or their role in Medical Admission Units, the usual admission route following ED [6, 7].

### Importance

Over the last 10 years there has been an expansion of United Kingdom (UK) Universities training pharmacists and there is now a surplus of registered pharmacists in England [8].

Since 2006 clinical pharmacists in UK have been able to undertake further training to become fully independent prescribers [9]. In March 2015 there were 2191 pharmacists with independent prescribing rights registered with the national regulatory body, the General Pharmaceutical Council. Clinical pharmacist independent prescribers may benefit from further training beyond prescribing, and are eligible to take a recently introduced educational programme to become Advanced Clinical Practitioners (ACP) [10]. Pharmacists with ACP training can conduct comprehensive physical examinations, request and interpret tests, diagnose and treat illnesses and injuries, and counsel patients on preventive healthcare. The proposed extended role for pharmacists with ACP training is not intended to replace the existing workforce, but to become a complementary group of clinicians who can diversify and become part of a fully integrated consultant-led team in the ED [11].

## Aim of the study

To determine the potential for pharmacists to clinically manage patients within ED; and from this investigation identify the clinical areas most likely to be impacted by extending the role of the pharmacists; and identify the training needs for the future ED workforce of pharmacists.

## Ethics approval

The study was commissioned and approved by Health Education England (West Midlands). The Research and Development departments of the data capture sites confirmed that the study was classified as service development, and therefore further approval was not required.

## Method

### Study design and setting

Multi-site, cross-sectional observational study conducted by independent prescribing pharmacist data collectors within 49 hospital sites in England (primary categorization). A range of hospitals from the regions of England participated in the study: Buckinghamshire and Oxfordshire (n = 4), East Anglia (n = 4), East Midlands (n = 4), London (n = 8), North East (n = 4), North West (n = 8), South (n = 2), South East (n = 4), South West (n = 5), West Midlands (n = 2), Yorkshire and Humber (n = 4). All hospitals were type 1 Emergency Departments—consultant led 24 hour service with full resuscitation facilities and designated accommodation for the reception of accident and emergency patients. Only two West Midland hospitals were included in the study as two other hospitals had already taken part in the pilot project that was used to validate and refine the methodology of data capture [12].

### Selection of participants

A representative sample of cases was taken from a cross-section of attendees and care pathways at each study site to reflect the usual workload characteristics of the departments. An independent prescriber pharmacist (IPP) based at the participating hospital was seconded from their usual role to undertake data capture within the ED at their site. Each IPP was asked to observe 400 ED attendee cases that would represent the typical case-mix of patients and record anonymized details of the case. Patients of all age ranges and presentations who attended ED at the time of the data collection were included in the study.

### Interventions

This study was non-interventional and was a cross-sectional observational study where pharmacists were observing a representative sample of ED attendees, and assessing each for the potential of the case to be clinically managed by pharmacists with or without advanced clinical practice training.

### Methods and measurements

Each data capture pharmacist was asked to record anonymized details of the cases and categorize each into one of four possible categories. They made this choice according to their own experience and knowledge of pharmacist training and range of competencies. The data capture instrument had been previously validated by a smaller pilot project based in the West Midlands [12]. An anonymized data-set for each attendee was recorded and managed using Microsoft Excel 2010 and a purpose built Microsoft Access database. Each site was requested to provide anonymized details of 400 cases.

The four categorizations were applied to each of the cases recorded during the study. These were:

- *CP, community pharmacist* could be managed by a community pharmacist (CP) working in a community pharmacy. (That is: attendance at ED was not necessary). CPs in the UK have at least 5 years training.
- *IP, independent prescriber pharmacist* could be managed by a hospital pharmacist with Independent Prescriber status. IPs have further post-registration training that gives them some clinical assessment skills and allows them to be fully independent prescribers. IPs have at least 8 years training/experience.
- *IPT, independent prescriber pharmacist with additional training* could be managed by a hospital pharmacist with Independent Prescriber status and additional clinical training, aligned to the Advanced Clinical Practice training pathway. The study was designed to identify what further training—in addition to the skills acquired from the General Pharmaceutical Council accredited independent prescribing course—would be most useful to support clinical management of ED attendees.
- *MT, medical team* unsuitable for pharmacist management—requires medical team management.

Primary categorization of harvested presentations was undertaken by the data capture independent prescriber pharmacists (IPPs, n = 63) at the study sites at the time of the presentation. These staff had access to the full patient details at the point of data capture. The pharmacists were chosen to do the primary review of cases, as they were the best placed health care professional with an understanding of the competencies of a pharmacist independent prescriber, and what additional skills they would need to extend their role.

### Outcomes

The primary outcome measure was the proportion of ED cases that could be managed by a pharmacist with or without further ACP training.

The secondary outcome measures:

- Identification of the clinical areas that are most likely to be impacted by extending the role of the pharmacist.
- Identification of training needs for future ED pharmacists—via content analysis of the IPT training needs described by the primary categorizer pharmacists.

### Analysis

All data were recorded on Microsoft Excel 2010, managed using Microsoft Access 2010 and exported into IBM SPSS version 21 and Minitab 17 for descriptive and statistical analysis.

Qualitative analysis of the IPT training needs specified by the primary categorizer was done via NVIVO version 10, and qualitative content analysis on the recorded text of training needs based on case observations [13]. The training need themes identified and coded from the recorded text, were allocated into one of four main categories: clinical examination and assessment, diagnosis skills, medical management, and treatment and training course components. The frequency of occurrences for each theme of training needs were determined. The training needs were coded by a trained research assistant (LM) and confirmed by a research pharmacist (CH) for consistency.

### Primary categorizations (on-site, by IPPs, in real-time)

Summary statistics for each category—CP, IP, IPT and MT were calculated.

### Secondary categorizations (off-site, by ED physicians, ED nurses and pharmacists)

Secondary categorization was undertaken by reference to the anonymized summary information recorded for this purpose by the data capture IPPs. The data-set included age, presenting complaint and clinical grouping. The clinical cases were recorded and randomised using a purpose designed Microsoft Access database. The database was programmed to generate 800 cases of ED attendees for each secondary categorizer to consider—who was asked to categorize each into the four categories defined within the study (CP, IP, IPT, MT). The primary categorization for the cases were removed prior to secondary categorization. Blind secondary categorization was undertaken by 15 pharmacists, 6 ED physicians and 4 ED nurses. This was completed personally by each of the secondary categorizers, without consultation, and without reference to categories assigned by others. Summary secondary categorization is expressed either as cases (based on a mean of all categorizations awarded for an individual case—type A calculation) or counts (summation of all categorizations—type B calculation).

The primary and secondary categorizations were compared and the level of agreement between the two identified across the four categories (presumed to be non-ordered/not ranked)—assessed using a kappa statistic via Minitab^®^ version 17. A non-ranked categorical kappa statistic was considered to be most appropriate in this case because, although there was increasing levels of training (community pharmacist least, through to medical team the most), the consistency of such differences are not clear.

### Regional variation

The categorization data for both the primary and secondary data was split into regions across England for regional variation analysis.

### Impact index

Cases were assigned a clinical grouping in relation to the nature of their admission. The clinical groupings were:

- Cardiology
- Ear nose and throat
- Gastroenterology
- Gynaecology
- Medicine—general
- Neurology
- Obstetrics
- Oncology
- Orthopaedics
- Psychiatry
- Respiratory
- Surgery—general
- Urology
- Other

An impact index score was calculated for each clinical grouping. This provides a measure of the potential for pharmacists to support the clinical workload in that grouping. The impact index algorithm accommodates both the workload associated with the clinical group (frequency of presentation) and the potential proportion of patients that may be managed by pharmacists.

The impact index was calculated as:

$${\text{Impact}}({\text{i)}} = \frac{{{\text{Total}}\,{\text{cases}}\,{\text{of}}\,{\text{CP}},{\text{IP}},{\text{IPT}}\,{\text{in}}\,{\text{the}}\,{\text{clinical}}\,{\text{group}}}}{{{\text{Total}}\,{\text{number}}\,{\text{of}}\,{\text{cases}}\,{\text{in}}\,{\text{the}}\,{\text{clinical}}\,{\text{group}}}} \times \frac{{{\text{Total}}\,{\text{number}}\,{\text{of}}\,{\text{cases}}\,{\text{per}}\,{\text{clinical}}\,{\text{group}} }}{{\begin{array}{*{20}c} {{\text{total}}\,{\text{number}}\,{\text{of}}\,{\text{cases}} } \\ {({\text{excluding}}\,{\text{those}}\,{\text{where}}\,{\text{clinical}}\,{\text{grouping}}\,{\text{was}}\,{\text{not}}\,{\text{assigned}})} \\ \end{array} }}$$

The algebraic expression is:

Impact (i) = proportion of workload of grouping (w) × proportion ability of pharmacists to manage that clinical group (a) × 100

$$I(i) = w \times a \times 100$$

The higher the Impact Index the greater potential for pharmacists to support the clinical workload in that group presenting to EDs.

Clinical grouping is not fully synonymous with the usual case mix of clinical specialties, but rather is a subset of Emergency Department attendees. Clinical grouping is used in this study to group presentations to identify clinical areas suitable for inclusion in advanced clinical practice training.

## Results

18,613 ED cases were observed from 49 sites between March to July 2015. The age of cases ranged from 0–115 years with a median age of 44 years and mode age of 27 years (interquartile range 27–67 years). The results of the categorization of cases from the primary data collection independent prescriber pharmacists are shown in Table 1 below.

**Table 1:** Summary of the proportion of cases that underwent primary categorizations and counts during secondary categorization—type B, per category

	Cases (primary categorization)	%	Counts (secondary categorization—type B)	%
CP	726	3.90	479	2.40
IP	719	3.90	1784	8.90
IPT	5202	27.90	4937	24.70
MT	11,966	64.30	12,777	64.00
Total	18,613	100	19,977	100
Total pharms	6647	35.70	7200	36.04

*CP* community pharmacist, *IP* independent prescriber pharmacist, *IPT* independent prescriber pharmacist with additional training, *MT* medical team only

The total number of cases that can be managed by a pharmacist (including cases where pharmacists specified what additional training beyond that currently undertaken by independent prescriber pharmacists—category IPT) is 6647 (35.7%).

### Secondary categorization

Secondary categorizations were undertaken by pharmacists (n = 15), ED physicians (n = 6) and ED nurses (n = 4); results are shown as ‘counts’ in Table 1 above. By design a single case may be assigned for secondary categorization more than once, and by any combination of pharmacists, physicians or ED nurses. During the study a total of 13,990 cases were secondary categorized at least once, which was undertaken a total of 19,977 times (described as ‘counts’).

Table 1 shows the sum of all secondary categorizations expressed in the four categories (CP, IP, IPT, MT)—calculation type B (counts)

Table 2 below shows secondary categorization by cases—calculation type A (cases).

**Table 2:** Summary of cases that underwent secondary categorization per category

	Cases	%
CP	246	1.80
CP/IP	33	0.20
IP	794	5.70
IP/IPT	339	2.40
IPT	3716	26.60
IPT/MT	828	5.90
MT	8034	57.40
Total	13,990	100
Total pharms	5128	36.70

These are mean values as some cases were categorized by more than one secondary categorizer (calculation type-A)

*CP* community pharmacist, *IP* independent prescriber pharmacist, *IPT* independent prescriber pharmacist with additional training, *MT* medical team only

Secondary categorization, using mean per case [*calculation* (*type A*)*: award numerical values to ordinal data* (*CP* = *1*, *IP* = *2*, *IPT* = *3*, *MT* = *4*)*. Take mean of score for each case*, *and express each case to one of 7 categories* (*CP*, *CP/IP*, *IP*, *IP/IPT*, *IPT*, *IPT/MT*, *MT*)].

The most frequent clinical groupings were: general medicine (36.4%), orthopaedics (16.5%), cardiology (5%), general surgery (4.9%) and respiratory (4%).

The clinical groupings where pharmacists can potentially have the highest impact are listed in Table 3 below.

**Table 3:** Top 5 impact index clinical groupings determined from the primary categorizations (clinical groups that will be impacted the most by having pharmacist roles extended through ACP training)

	Total cases	Total cases ∑CP + IP + IPT	Impact index
Medicine-general	6774	2212	13.2
Orthopaedics	3072	1627	9.7
Respiratory	751	308	1.8
Ear, nose and throat	513	276	1.6
Gastroenterology	723	212	1.3

The level of agreement between the primary categorizers (Pharmacist Independent prescriber data collectors) and physician secondary-categorizers was calculated on a case by case basis. A kappa statistic of 0.26 (standard error 0.011, with a 95% confidence interval of 0.24–0.28) was obtained—indicating a fair agreement. See Table 4 for a cross-tabulation of the primary versus doctor secondary-categorizer. Exact agreement of the category on a case-by-case basis was obtained in 60.1% of cases. If category is considered as ordered, disagreement by more than one category occurred in just 10.6% of cases. Overall the total cases categorized to a pharmacist (sum of CP, IP and IPT) by secondary categorization physicians was 1667 from 4421 (37.7%)—primary categorization of the same cases to a pharmacist category is 1659 (37.5%).

**Table 4:** Comparison of the pharmacist primary and doctor secondary-categorizations of cases

	Secondary physician categorization				
	CP	IP	IPT	MT	Total
*Primary pharmacist categorization*					
CP	35	53	43	38	169
IP	21	64	45	75	205
IPT	65	183	476	561	1285
MT	62	186	434	2080	2762
Total	183	486	998	2754	4421

### Regional analysis of the data

The categorization data in different regions varied concerning pharmacists’ potential to manage ED patients, with results ranging from 16.1 to 43%—with the West Midlands being a clear outlier according to their primary categorization.

The Pearson Chi square test comparing pharmacist-categories (combined) across regions gave a value of 365.533, *DF* = 10, with a *p* value <0.001.

**Fig. 1:**
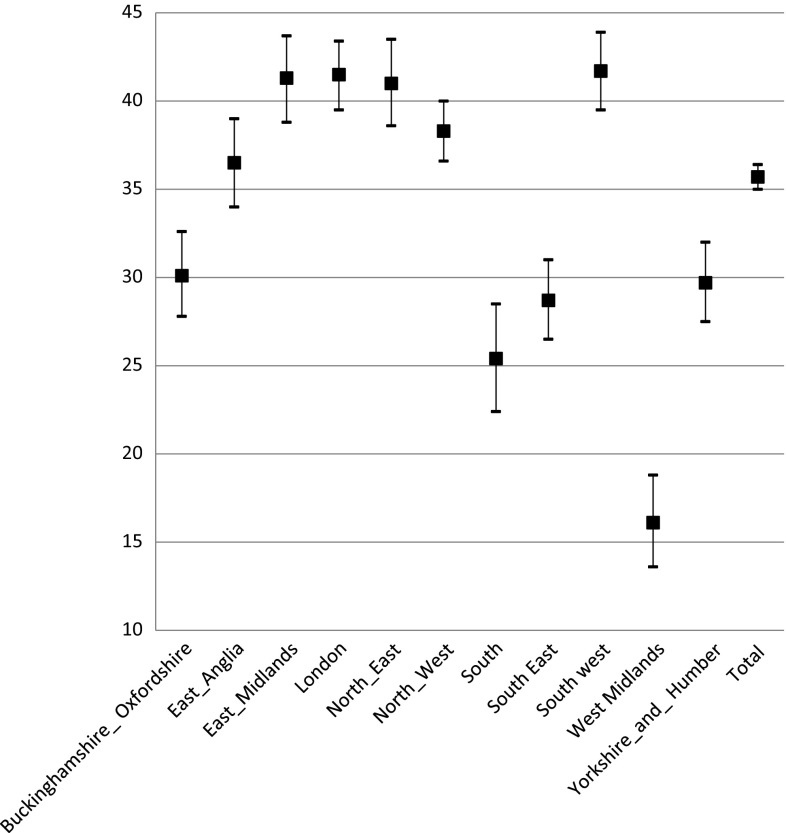
Primary categorization percentage of cases manageable by the pharmacist (with 95% CI)

**Fig. 2:**
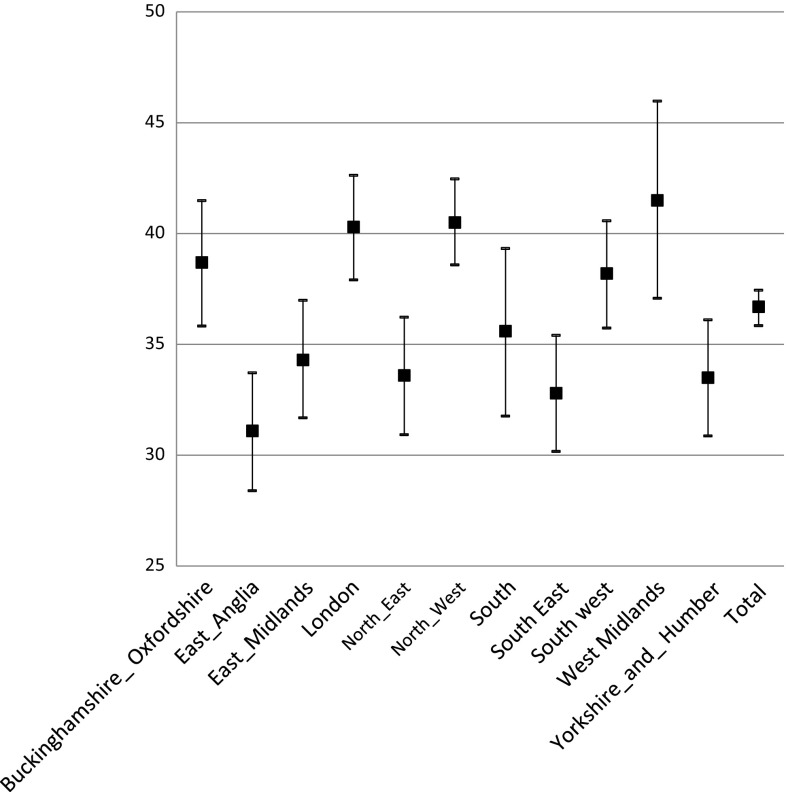
Secondary categorization percentage of cases manageable by the pharmacist (with 95% CI)

 Table 5 below shows the summary regional data. Figures 1 and 2 shows the percentage of ED cases that were deemed manageable by a pharmacist per region (primary-categorization and secondary-categorization respectively).

**Table 5:** Regional variations in the primary and secondary categorization of ED attendees (49 sites)

Region	Number of sites	Number of cases per region	Primary category					Number of cases per region	Secondary category (calculation type B)				
			CP (%)	IP (%)	IPT (%)	Pharmacist combined [% (95% CI)]	MT (%)		CP (%)	IP (%)	IPT (%)	Pharmacist combined [% (95% CI)]	MT (%)
Buckinghamshire Oxfordshire	4	1470	4.2	3.9	22.0	**30.1** (27.8–32.6)	69.86	1138	2.1	6.2	30.3	**38.7** (35.83–41.49)	61.34
East Anglia	4	1484	2.6	1.8	32.1	**36.5** (34–39)	63.54	1159	1.6	4.1	25.4	**31.1** (28.4–33.72)	68.94
East Midlands	4	1600	2.8	5.9	32.6	**41.3** (38.8–43.7)	58.75	1229	1.1	4.6	28.6	**34.3** (31.69–36.99)	65.66
London	8	2480	10.0	8.0	23.4	**41.5** (39.5–43.4)	58.55	1654	3.3	7.3	29.7	**40.3** (37.91–42.63)	59.73
North East	4	1595	0.6	0.6	39.9	**41.0** (38.6–43.5)	59.00	1224	1.1	6.2	26.3	**33.6** (30.93–36.23)	66.42
North West	8	3200	2.0	3.6	32.7	**38.3** (36.6–40.0)	61.75	2470	1.8	7.2	31.6	**40.5** (38.59–42.47)	59.47
South	2	800	1.6	6.0	17.8	**25.4**(22.4–28.5)	74.63	616	1.0	6.5	28.1	**35.6** (31.77–39.33)	64.45
South East	4	1600	1.3	1.4	26.0	**28.7** (26.5–31.0)	71.31	1229	1.1	5.9	25.7	**32.8** (30.17–35.41)	67.21
South West	5	2000	4.6	4.2	33.0	**41.7** (39.5–43.9)	58.30	1554	1.0	4.6	32.6	**38.2** (35.74–40.58)	61.84
West Midlands	2	784	2.4	2.2	11.5	**16.1** (13.6–18.8)	83.93	472	2.8	4.9	33.9	**41.5** (37.08–45.98)	58.47
Yorkshire and Humber	4	1600	7.4	2.9	19.4	**29.7** (27.5–32.0)	70.31	1245	2.6	5.6	25.3	**33.5** (30.87–36.11)	66.51
Total	49	18,613	3.9	3.9	27.9	**35.7** (35–36.4)	64.29	13,990	1.76	5.91	28.98	**36.7** (35.85–37.45)	63.35

### Training needs content analysis

The training needs described by the primary categorizers (primary categorizers from 46 sites provided suggestions as free text) were considered using content analysis, and four main themes were identified. These are:

1. Clinical examination and assessment (42 sites, n = 4510)
2. Diagnostic skills (36 sites, n = 1381)
3. Medical management and treatment (46 sites, n = 1236)
4. Training course component (16 sites, n = 359)

The most frequent top 10 subthemes for each of the four mains themes are described in Tables 6, 7, 8 and 9 below.

**Table 6:** Clinical examination and assessment (42 sites, n = 4510)

Subtheme (top 10)	Number of categorizers involved in providing training needs information	Number of times suggested (n)
1. X-ray request and interpretation	31	1428
2. Body examination (e.g. external body)	37	959
3. Clinical examination and assessment	12	295
4. Clinical skills	2	266
5. Neurological assessment	20	220
6. Paediatrics	17	137
7. Chest examination	27	132
8. Respiratory assessment or examination	15	93
9. Eye examination	18	92
10. Observations	5	76

**Table 7:** Diagnostic skills (36 sites, n = 1381)

Subtheme (top 10)	Number of categorizers involved in providing training needs information	Number of times suggested (n)
1. ECG	23	546
2. Bloods	14	426
3. Urine testing	10	258
4. Arterial blood gas interpretation	4	22
5. Differential diagnosis	4	20
6. Troponin T	4	12
7. D-dimer test request	4	11
8. CT Scan interpretation	2	7
9. Blood pressure	5	6
10. Doppler	2	5

**Table 8:** Medical management and treatment (46 sites, n = 1236)

Subtheme (top 10)	Number of categorizers involved in providing training needs information	Number of times suggested (n)
1. Trauma and injury management	14	136
2. Wound care	16	109
3. Analgesia	3	107
4. Paediatric management	13	62
5. Fracture management	7	57
6. Minor illnesses	3	42
7. Pain management	7	37
8. Nosebleeds	7	33
9. Respiratory treatment	7	33
10. Skin conditions	7	32

**Table 9:** Training course components (16 sites, n = 359)

Subtheme	Number of categorizers involved in providing training needs information	Number of times suggested (n)
1. Minor injuries course	14	316
2. Radiology	1	41
3. Dermatology clinical skills	1	1
4. Knowledge of compartment syndrome	1	1

## Discussion

This study found that approximately 1 in 13 of recorded ED attendee cases (7.8%) were considered manageable by pharmacists with existing training (CPs and IPs combined). This rises to a maximum of 36% if pharmacists are given further advanced clinical practice training (CP, IP and IPT combined) as identified by the study respondents.

An additional outcome measure evaluated during the course of the study was which particular clinical groups within the ED would benefit from having a pharmacist manage that group, based on the calculated Impact Index. The Impact Index was defined to be sensitive to both the pharmacist’s potential to manage the cases, and the workload of that group. General Medicine and Orthopaedics were found to have the highest Impact Index. To enable pharmacists to best support the clinical workload of ED further advanced clinical practice training should focus on these two clinical areas, and if achieved would enable Advanced Clinical Practitioner Pharmacists (ACP-Pharmacists) to manage 27% of all ED attendees.

This study is the first published work to evaluate the potential of future ACP-Pharmacists within EDs—working beyond traditional clinical pharmacist roles. This study estimates for the first time the proportion of ED attendees ACP-Pharmacists may be able to manage, when situated within the ED, and as part of the multi-disciplinary team. Other clinical professions who have ACP status such as Advanced Nurse Practitioners (ANP) and Paramedic Practitioners (PP) have a long establish role in clinical practice, however the body of published evidence supporting these roles is limited. The only published study which has evaluated the proportion of cases that ANPs can manage (one hospital site over 8 months) found that 20% of all ED cases classified as ‘minor’ (1577/7768) could be managed by the nurse [14]. The evidence base for PPs showed via a randomized controlled trial that they were key in preventing hospital admission to ED for older adults by being part of the ambulance team [15]. To date there are no published studies considering the role of PPs working within the ED.

This study builds on and adds generalizability to the previous pilot study conducted in the West Midlands. The pilot study found that 45% of ED attendees were potentially manageable by pharmacists across the two study sites [12], which is higher than the overall findings of this present national study (36%). However, the proportion of cases that were considered manageable by pharmacists with current training (CPs and IPs) was 1 in 13 for this study, which is similar to the pilot study proportion of 1 in 12 [12].

The results show that the proportion of cases deemed to be manageable by pharmacists according to primary categorization vary from region to region—range 16.1–41.7%, mean 35.7% (95% CI 35.0–36.4%). Interestingly secondary categorization gives a narrower range—31.1–41.5%, mean 36.7% (95% CI 35.85–37.45%), which may indicate an outlier amongst the primary categorizers. Although pharmacist independent prescribers have the same qualification and must have at least 2 years post-registration experience, this does not fully standardize the data categorization across the 49 sites. Years of experience prior to becoming a prescriber may vary.

Current literature indicates that the work of hospital pharmacists in the EDs of the UK have been limited to traditional pharmacy roles. These include: medicines reconciliation for high risk patients, transcription of drug charts, increasing patient-own-drugs use, reviewing medication and direct support of transfer of changed medicines information on discharge from the ED, e.g. liaising with the family physician, community pharmacist and care homes [16]. In the United States of America (USA), the roles of the pharmacist in the ED are a collaborative supportive role and mainly involve medicines management, medicines reconciliation, educating and counseling patients and carers on safe and effective use of their medication. They provide direct patient care, but only as part of the interdisciplinary emergency care team [16, 17]. Our study suggests that pharmacists with additional training can undertake clinical management of a wide range of patients as part of the multi-disciplinary ED workforce.

## Limitations

Due to the size of the study, it was not feasible in terms of resources to conduct the secondary categorization by direct observation of each case prior to making a clinical judgement. However 75% of cases were categorized twice (both primary and secondary categorization). The method of secondary categorization (summary anonymized data) was validated in a previous pilot study. This present study recruited 63 pharmacist independent prescribers to observe cases within the ED and judge whether these were manageable by a pharmacist (n = 18,613, 400 cases per site). For sites with more than 1 independent prescriber data collector, the 400 cases were shared across the data collectors, with one data collector per observation. It would not have been feasible to ask the data collectors to see the patients and manage them in real time. Future studies may include identifying the contribution Advanced Clinical Practitioner pharmacists can actually make towards managing the workload in EDs once training has been completed; and the in-practice experience needed to ensure clinical competence.

This study did not assess in detail cases managed by the medical team that could not be resolved without the need of a clinical contribution by a pharmacist, e.g. cases where the medical team would contact a pharmacist for advice to resolve a case.

## Conclusion

In summary, primary categorization of 18,613 ED cases confirms the potential for pharmacists (all categories) to clinically manage up to 36% of ED attendees. With existing training (CPs and IPs) pharmacists can potentially manage 8% of ED cases. Further training aligned to the Advanced Clinical Practice training pathway (IPTs—Advanced Clinical Practice Pharmacists) increases the potential of pharmacists to manage a further 28% of cases. Secondary categorization of the data (a total of 75%, n = 13,990) supports the validity of the primary categorization findings. Impact Index calculations suggest that pharmacists with advanced training (IPTs—Advanced Clinical Practice pharmacists) may be most usefully trained in general medicine and orthopaedics/minor trauma. If training were to concentrate on the two areas with the highest Impact Index (probably achievable with 12 months advanced clinical training) then, (achievable) IPT becomes 19%, i.e. pharmacists overall could manage 27% of cases attending ED. In order for pharmacists to manage cases, they require clinical examination assessment skills as well as diagnostic skills to broaden and extend their role and case management potential.
